# Considerable differences in the utilisation of antidiabetics between Serbia and Scandinavian countries

**DOI:** 10.1186/2050-6511-13-S1-A6

**Published:** 2012-09-17

**Authors:** Nebojša Pavlović, Milica Paut Kusturica, Bojan Stanimirov, Maja Stojančević, Ana Sabo, Momir Mikov

**Affiliations:** 1 Department of Pharmacology, Toxicology and Clinical Pharmacology, Faculty of Medicine, University of Novi Sad, 21000 Novi Sad, Serbia

## Background

Diabetes mellitus is a major public health concern with devastating human, social and economic impact. It is increasing globally, affecting more than 180 million people worldwide. The objective of our study was to analyse the overall volume of use of antidiabetics in Serbia compared to Scandinavian countries (Sweden, Norway, Denmark), chosen for their rational and conservative prescription practice.

## Methods

Data on consumption of antidiabetics (ATC group A10) in 2010 were extracted from the databases of the representative national authorities. Utilisation of these medicines was measured through the defined daily dose (DDD) unit and the results were expressed as DDD per 1000 inhabitants per day (DID).

## Results

In 2010, antidiabetics were used at a similar rate in Serbia (47.3 DID) and Scandinavian countries (from 46.5 DID in Sweden to 47.67 DID in Norway), but the share of use of insulins (A10A) and oral antidiabetics (A10B) differed among the observed countries. The proportion of insulin in Serbia was 22.0% of all antidiabetics which is relatively low in comparison with Scandinavian countries (from 36.2% in Denmark to 50.8% in Sweden). Utilisation of long-acting insulins (A10AE) was much lower in Serbia (1.3 DID) compared to Scandinavian countries (range: 2.6–4.6 DID). The share of oral antidiabetics use also differed among these countries. In Serbia, sulfonylureas (A10BB), as a second-line treatment for type 2 diabetes, were used predominantly (55.6%) compared to metformin (44.1%). In Scandinavian countries, metformin, as preffered oral agent for type 2 diabetes and the only medicine from the biguanide class (A10BA), was used at a higher rate than in Serbia (from 51.2% in Denmark to 60.4% in Sweden). New medicinal products with effect on the incretin system (A10BH and A10BX) were also used at a higher rate in Scandinavian countries (range: 0.5–2.5 DID) in comparison to Serbia (0.002 DID).

## Conclusions

This cross-national study has demonstrated large differences in the utilisation of various antidiabetics among observed countries that may be attributed to considerable variations in attitudes and habits, especially with regard to the management of type 2 diabetes.

